# The Impact of the Angiotensin-Converting Enzyme Inhibitor Lisinopril on Metabolic Rate in *Drosophila melanogaster*

**DOI:** 10.3390/ijms251810103

**Published:** 2024-09-20

**Authors:** Denise Vecchie’, Julia M. Wolter, Jesse Perry, Patricia Jumbo-Lucioni, Maria De Luca

**Affiliations:** 1 Department of Nutrition Sciences, University of Alabama at Birmingham, Birmingham, AL 35294, USA; dvecchie@uab.edu (D.V.); jmwolter@uab.edu (J.M.W.); jcperry@uab.edu (J.P.); 2 Department of Pharmaceutical, Social and Administrative Sciences, Samford University, Birmingham, AL 35229, USA; pjumbolu@samford.edu; 3 Department of Biology, University of Alabama at Birmingham, Birmingham, AL 35294, USA

**Keywords:** oxygen consumption rate, ACE inhibitors, bradykinin, aging, losartan, brain, Malpighian/renal tubules

## Abstract

Evidence suggests that angiotensin-converting enzyme inhibitors (ACEIs) may increase metabolic rate by promoting thermogenesis, potentially through enhanced fat oxidation and improved insulin. More research is, however, needed to understand this intricate process. In this study, we used 22 lines from the *Drosophila* Genetic Reference Panel to assess the metabolic rate of virgin female and male flies that were either fed a standard medium or received lisinopril for one week or five weeks. We demonstrated that lisinopril affects the whole-body metabolic rate in *Drosophila melanogaster* in a genotype-dependent manner. However, the effects of genotypes are highly context-dependent, being influenced by sex and age. Our findings also suggest that lisinopril may increase the *Drosophila* metabolic rate via the accumulation of a bradykinin-like peptide, which, in turn, enhances cold tolerance by upregulating *Ucp4b* and *Ucp4c* genes. Finally, we showed that knocking down *Ance*, the ortholog of mammalian ACE in Malpighian/renal tubules and the nervous system, leads to opposite changes in metabolic rate, and that the effect of lisinopril depends on *Ance* in these systems, but in a sex- and age-specific manner. In conclusion, our results regarding *D. melanogaster* support existing evidence of a connection between ACEI drugs and metabolic rate while offering new insights into this relationship.

## 1. Introduction

**Figure 1:**
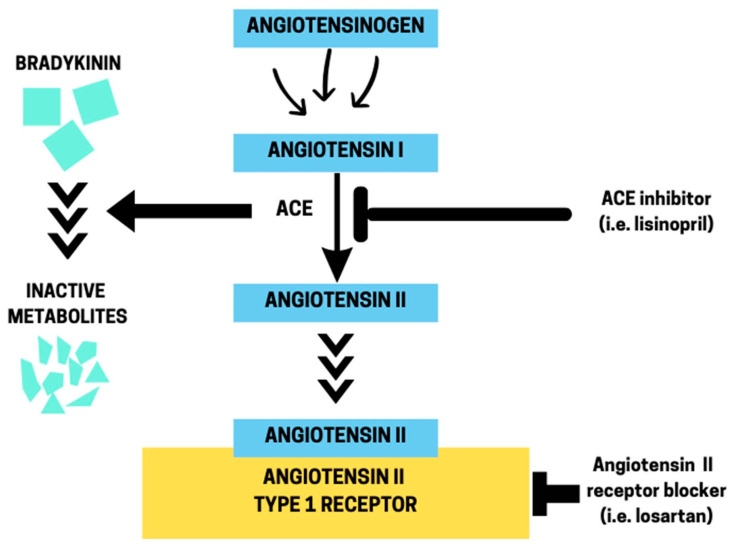
Dual role of angiotensin-converting enzyme (ACE). ACE converts Angiotensin I into Angiotensin II and degrades bradykinin. The drug lisinopril inhibits ACE, whereas losartan antagonizes the binding of Angiotensin II to its type-1 receptor.

The renin–angiotensin system (RAS) is a complex physiological system that is implicated in the control of blood pressure and fluid and electrolyte balance [1]. An evolutionarily conserved component of RAS is the angiotensin-converting enzyme (ACE), which plays a dual role in maintaining blood pressure by converting angiotensin (Ang) I into the active vasoconstrictor Ang II and degrading bradykinin, a vasodilatory and natriuretic peptide [2] (Figure 1). Ang II exerts its actions primarily through binding to two main types of receptors, type-1 receptor (AT_1_R) and type-2 receptor (AT_2_R), which are found in various tissues throughout the body, where they have opposite effects on vascular tone [1]. On the other hand, bradykinin works through the stimulation of B_2_ receptors that are present on endothelial cells [3]. ACE inhibitors (ACEIs), such as lisinopril, are the most frequently prescribed medications in the US to treat hypertension and manage heart failure, post-myocardial infarction, and chronic kidney disease [4]. However, ACEIs are also highly beneficial in hypertensive patients with type-2 diabetes due to their interactions with systems and molecules involved in glucose and lipid metabolism [5]. Growing evidence indicates that the impact of ACEIs extends beyond these metabolic processes. In this regard, animal studies have revealed a tight link between RAS components and an organism’s metabolic rate. For instance, global knockout of *Ace* in mice resulted in small and lean animals that exhibited increased energy expenditure and lipolysis [6]. Likewise, *angiotensin II receptor*, *type 1a* (*Agtr1a*) knockout rats had higher energy expenditure and fatty acid oxidation in adipose tissue than control rats, following a high-fat diet for 12 weeks [7]. However, it has also been reported that the hyperactivation of RAS, specifically in the brain of mice, results in a marked increase in body temperature and metabolic rate through its direct stimulation of the sympathetic nervous system and consequent activation of adipose tissue thermogenesis [8]. These observations indicate that the control of whole-organism metabolic rate by RAS components and their inhibitors is multifaceted, highlighting the need for further research to fully understand this intricate process.

Lately, *D. melanogaster* has become an effective model to investigate the relationship between ACEI therapy and metabolism for several reasons. First, many genetic and genomic resources have been developed and are publicly available, including the *Drosophila* Genetic Reference Panel (DGRP), a resource of fully sequenced inbred lines that are currently available to the scientific community [9,10]. Second, *D. melanogaster* has two distinct homologs of human *ACE*, called *Ance* and *Angiotensin-converting enzyme-related* (*Acer*), that encode active enzymes [11,12]. Earlier work showed that the enzymatic activity of Ance is inhibited by the same drugs that inhibit human ACE, such as lisinopril, through a similar mechanism [13]. Third, although Ang II and its receptors have not been identified in *D. melanogaster* [14], earlier studies reported that losartan, an angiotensin receptor blocker (ARB), significantly rescued memory deficits in a *Drosophila* model of Alzheimer’s disease (AD) [15] and improved mitochondrial morphology in indirect flight muscles of *Drosophila* mutants of *Multiplexin*, the ortholog of vertebrate collagen XV and XVII [16]. These observations strongly suggest that a potential counterpart of the vertebrate Ang II/AT_1_ receptor system might be present in *D. melanogaster*. Finally, invertebrates like *D. melanogaster* do not have a vasculature system, thereby making it possible to disentangle the vascular hemodynamic effects of RAS blockade drugs from their direct impact on cellular metabolism.

Previously, we showed that lisinopril treatment impacts *Drosophila* thoracic mitochondrial and metabolic parameters in male flies [17], also providing evidence for the existence of a link between ACEIs and metabolic processes in flies. The main objective of this study was to assess whether lisinopril influences whole-body metabolic rate in both female and male *D. melanogaster* and if there is individual variability in response to the drug. To achieve this, we measured the metabolic rate in virgin female and male flies from a subset of the DGRP lines. Since we were also interested in assessing whether old age might influence the response to the drug, metabolic rate was assessed in young and old flies. We report that lisinopril treatment displays significant genotype-, sex-, and age-dependent effects on whole-body metabolic rate in *D. melanogaster*. We also reveal that the changes to the metabolic rate induced by lisinopril in these lines are not explained by changes in locomotor behavior. Further, we provide evidence that lisinopril influences metabolic rate through mechanisms that involve the reduction of an ancestral Ang II-equivalent signaling reduction and the accumulation of a bradykinin-like peptide. Finally, we show that the lisinopril effect on the whole-body metabolic rate depends on *Ance* expression in the Malpighian/renal tubules and the nervous system.

## 2. Results

### 2.1. Genotype, Age, and Sex Affect the Interindividual Changes in Metabolic Rate in Response to Lisinopril

We measured the metabolic rate as whole-body oxygen consumption rate in individual female and male flies from 22 DGRP lines at one week (young flies) and five weeks (old flies) of age. The lines were chosen to encompass, as much as possible, the genetic variation present in the entire panel. After adjusting for body weight, we analyzed our data using a four-way mixed analysis of variance (ANOVA), including the fixed effects of lisinopril treatment, sex, and age, the random effect of genotype, and all possible interaction terms. Our analysis revealed statistically significant effects of sex and age on metabolic rates (Table 1), with males exhibiting higher metabolic rates than females (males: 35.92 ± SE 0.37; females: 32.78 ± 0.33), and with old flies having lower metabolic rates than young flies (young: 36.19 ± 0.35; old: 32.54 ± 0.36). We did not observe significant effects of genotype or treatment; however, there were statistically significant effects for genotype-by-treatment, genotype-by-treatment-by-age, and genotype-by-treatment-by-sex-by-age interaction terms (Table 1).

**Figure 2:**
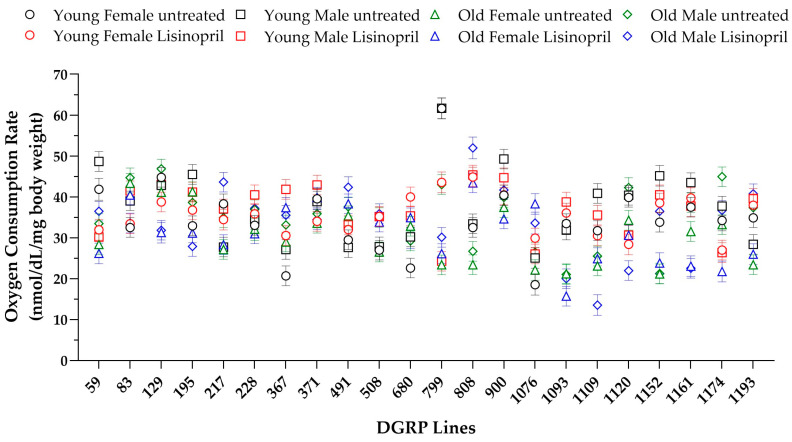
Changes in metabolic rate induced by lisinopril treatment in 22 DGRP lines. The response to lisinopril is influenced by genotype, sex, and age. Data report the means ± standard error of whole-body oxygen consumption rate adjusted for live body weight (*n* = 8–10 individual flies).

Thus, genetics contributes to the lisinopril-induced changes in metabolic rate, but the effects of genotypes are highly context-dependent, as they are also influenced by sex and age (Figure 2).

**Figure 3:**
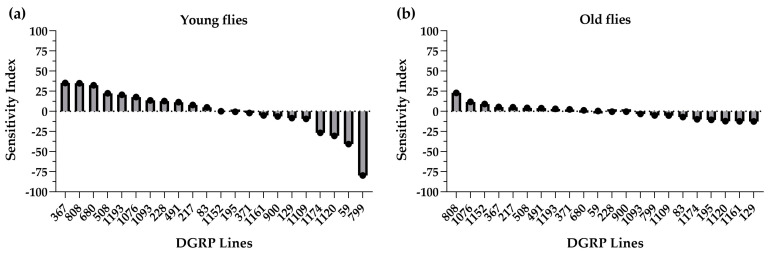
Sensitivity of metabolic rate to lisinopril. (**a**,**b**) Values represent sensitivity index in DGRP young (panel (**a**)) and old (panel (**b**)) flies, averaged over sex. Sensitivity indices were calculated by taking the difference in the average oxygen consumption rate between lisinopril-treated and untreated flies of each genotype and dividing it by the average difference in treated and untreated flies across all genotypes. Lines are ranked by their sensitivity, with positive values indicating a higher metabolic rate with lisinopril, relative to untreated and negative values indicating a lower metabolic rate.

To inspect the genotype-specific response of oxygen consumption rate to lisinopril at each age, we calculated the sensitivity index of Falconer [18] using the data averaged over both sexes. As previously seen by Gabrawy et al. for physical performance [19], we found that the direction and magnitude of the response to lisinopril depend on the genotypes at both ages (Figure 3). However, the range of sensitivity to the drug is larger in young flies (35.32 to −79.76; Figure 3a) than in old flies (22.96 to −12.61; Figure 3b), suggesting that the effects of genetic variation on changes in metabolic rate in response to lisinopril might become less important with advancing age.

### 2.2. Lisinopril Does Not Change the Metabolic Rate by Altering Locomotor Activity in DGRP Lines Displaying the Greatest Drug Response

**Figure 4:**
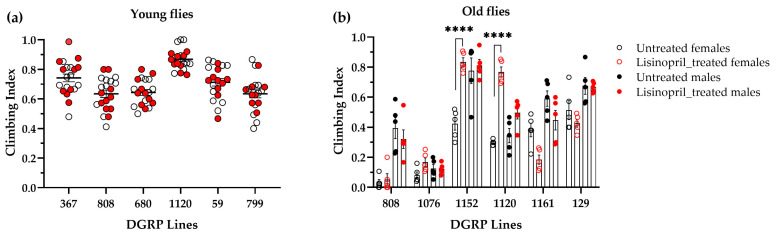
Lisinopril does not alter the metabolic rate through changes in locomotor activity. (**a**) There is no significant effect of lisinopril on the climbing ability of DGRP lines displaying the greatest change in metabolic rate in response to lisinopril at one week of age. Data report climbing indices for untreated (white dots) and lisinopril-treated (red dots) flies averaged over both sexes and ages. (**b**) The effect of lisinopril on the climbing ability of DGRP lines displaying the greatest change in metabolic rate in response to lisinopril at five weeks of age depends on genotype and sex. **** *p* < 0.0001 after post hoc Tukey’s tests for multiple comparisons. In both panels, error bars represent standard errors.

Next, we investigated whether lisinopril influences the metabolic rate through changes in locomotor activity. For this set of experiments (and henceforth), we selected the DGRP lines that showed the highest sensitivity to the drug at young and old age, independent of sex. In young flies, a three-way ANOVA revealed a significant main effect of sex (climbing index for males: 0.75 ± SE 0.012; females: 0.66 ± 0.012; F_1,96_ = 14.40, *p* = 0.0127) and a marginal effect of genotype (F_5,96_ = 13.43, *p* = 0.0627), but no effect of treatment or interaction terms (Figure 4a). In old flies, we did not find significant main effects of sex and treatment. However, there was a marginal effect of genotype (F_5,96_ = 4.02, *p* = 0.0509) and a significant effect for the sex-by-genotype-by-treatment interaction term (F_5,96_ = 4.50, *p* = 0.0010), with only lisinopril-fed DGRP_1120 and DGRP_1152 female flies exhibiting significantly different climbing activity than those untreated (Figure 4b). Of these two lines, lisinopril-fed DGRP_1152 female flies had both higher metabolic rate and climbing activity than untreated flies. In contrast, lisinopril-fed DGRP_1120 female flies displayed lower metabolic rate and higher climbing activity.

Thus, alterations in locomotor behavior do not explain the lisinopril-induced effects on metabolic rate in these lines.

### 2.3. Lisinopril Influences Drosophila Metabolic Rate through Mechanisms That Involve the Reduction of Ang II-Equivalent Signaling and Accumulation of a Bradykinin-like Peptide

**Figure 5:**
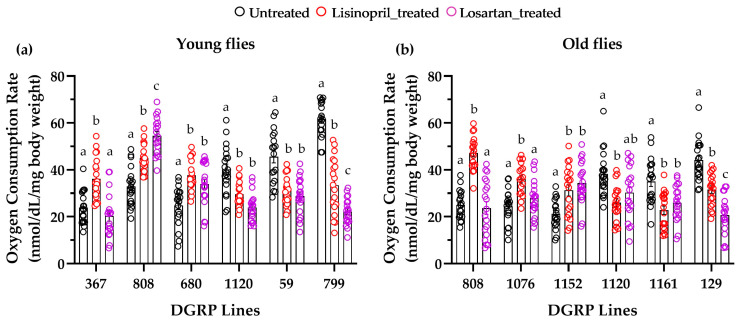
Losartan affects metabolic rate in *D. melanogaster*. (**a**,**b**) DGRP lines exhibiting the greatest change in metabolic rate in response to lisinopril in young or old flies received losartan for one week (panel (**a**)) and five weeks (panel (**b**)), respectively. In both panels, data are averaged over both sexes and error bars represent standard errors. Significant comparisons within each DGRP line were determined by post hoc Tukey’s tests at *p* < 0.05 and are indicated by different letters.

In mammals, the pharmacological effects of ACEIs are mediated via both the reduction in angiotensin II signaling and the accumulation of bradykinin [2]. To investigate whether it is possible to differentiate between the two mechanisms in *D. melanogaster*, we administered losartan, an Ang II receptor blocker (ARB) (see Figure 1), to flies of the DGRP lines mentioned above and then assessed the metabolic rate. Three-way ANOVAs revealed significant genotype-by-treatment interaction effects in young (F_10,312_ =13.87, *p* = 0.0001) and old (F_10,308_ = 6.79, *p* = 0.0028) flies. As shown in Figure 5, most of the lines responded to both lisinopril and losartan with significant changes in metabolic rate compared to untreated ones, with the direction of the response to both drugs being the same. However, while lisinopril treatment significantly increased the metabolic rate of DGRP_367 young flies (Figure 5a) and of DGRP_808 and DGRP_1076 old flies (Figure 5b), no differences were observed between those treated with losartan and their untreated counterparts. Of note, the effect of losartan on the metabolic rate of DGRP_1120 old flies is sex-specific, being present only in males (untreated: 42.30 nmol/dL/mg body weight ± 3.42 vs lisinopril: 21.97 nmol/dL/mg body weight ± 2.00, *p* < 0.0001; untreated vs losartan: 22.55 nmol/dL/mg body weight ± 2.65, *p* < 0.0001).

Thus, our results indicate that, depending on the genotype, lisinopril impacts the metabolic rate of both young and old flies through a mechanism that involves a reduction in an Ang II-equivalent signaling, the accumulation of a bradykinin-like peptide, or both.

### 2.4. Lisinopril Affects Acute Cold Tolerance in D. melanogaster in a Genotype-, Sex-, and Age-Specific Manner

**Figure 6:**
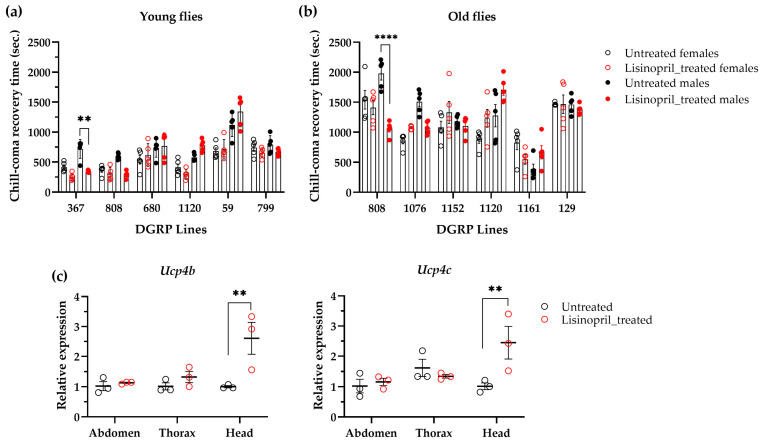
Effects of lisinopril on acute cold tolerance and UCP genes. (**a**,**b**) Acute cold tolerance in lisinopril-treated and untreated DGRP lines exhibiting the greatest change in metabolic rate in response to lisinopril at one week (panel (**a**)) and five weeks (panel (**b**)) of age. Lisinopril reduces time to recover from chill-induced coma only in DGRP_367 young males (panel (**a**)) and DGRP_808 old males (panel (**b**)). (**c**) The expression of *Ucp4b* and *Ucp4c* genes is significantly increased in the head of DGRP_367 young male flies following lisinopril treatment. Transcript levels of each target gene were normalized to *rp49* and *α-tubulin*. In all panels, ** *p* < 0.01 and **** *p* < 0.0001 after post hoc Tukey’s tests for multiple comparisons. Error bars represent standard errors.

It was recently reported that serum bradykinin is required to sustain the body temperature during acute cold tolerance in male mice [20]. The same study also showed that the administration of ACEIs increased bradykinin levels and the expression of brown adipose tissue *uncoupling protein-1* (*UCP1*) [20]. Based on these observations, we sought to investigate whether lisinopril might affect cold tolerance in *D. melanogaster* by measuring chill-coma recovery time (CCRT) in the subset of DGRP lines. In young flies, we observed a significant main effect of sex (males: 727.45 s ± SE 17.07; females: 500.52 s ± 17.07; F_1,96_ = 14.40, *p* = 0.0127) but no effect of genotype or treatment. However, there was a statistically significant genotype-by-sex-by-treatment interaction effect (F_5,96_ = 3.51, *p* = 0.0058), with lisinopril-treated DGRP_367 young males being 52% faster in recovering from chill-coma than untreated males (Figure 6a). While no significant effects of genotype, sex, or treatment were observed in old flies, a statistically significant effect of the genotype-by-sex-by-treatment interaction term (F_5,96_ = 5.78, *p* = 0.0001) was also detected. In this case, lisinopril-treated DGRP_808 old males were found to be 46% faster in recovering from chill-coma than untreated males (Figure 6b).

Cold-induced thermogenesis mediated by mitochondrial uncoupling proteins (UCPs) is also present in *Drosophila*, with UCP4B and UCP4C involved in the process [21,22,23,24]. To explore whether lisinopril might influence CCRT by changing the expression of UCP genes, we quantified the transcript levels of *Ucp4b* and *Ucp4c* in the abdomen, thorax, and head of DGRP_367 male flies that were either treated with lisinopril for one week or fed standard food. While there were no differences in *Ucp4b* and *Ucp4c* (Figure 6c) transcript levels between lisinopril-treated and untreated flies in both the abdomen and thorax, we observed a significant increase in the expression of both genes in the head of lisinopril-treated DGRP_367 male flies (Figure 6c).

Thus, based on our findings, we propose that, like in mammalian models, lisinopril might induce thermogenesis through the induction of UCPs in *D. melanogaster*.

### 2.5. The Effect of Lisinopril on Whole-Body Metabolic Rate Depends on the Expression of Ance in the Malpighian/Renal Tubules and the Nervous System

**Figure 7:**
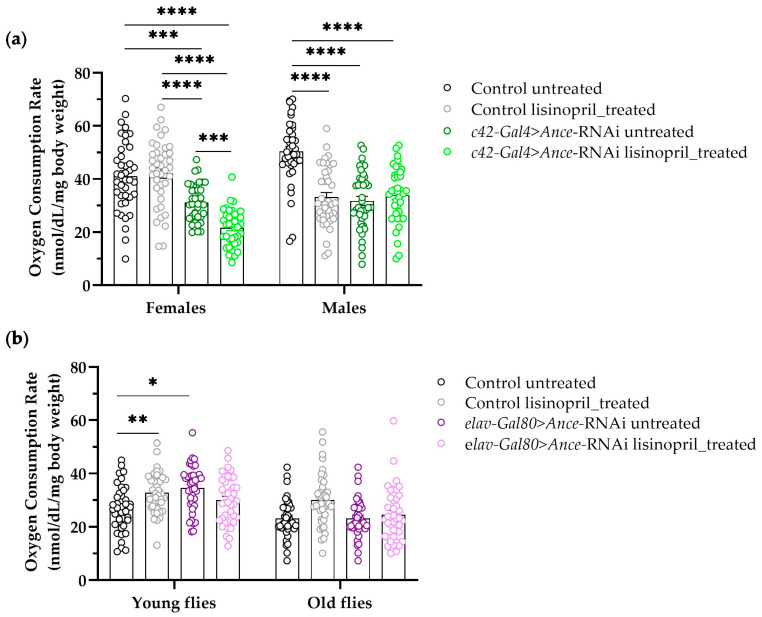
The effect of lisinopril on whole-body metabolic rate depends on *Ance* in an organ system-, sex-, and age-specific manner. (**a**) Metabolic rate of flies with *Ance* knocked down in Malpighian tubules (*c42*-*Gal4* > *Ance*-RNAi) and of their corresponding *w*^1118^ isogenic line (control). A four-way ANOVA revealed a significant genotype-by-treatment-by-sex interaction effect on oxygen consumption rate (F_1,301_ = 47.40, *p* < 0.0001). (**b**) Metabolic rate of flies with *Ance* knocked down in the nervous system (*elav*-*Gal80* > *Ance*-RNAi) and of their corresponding *w*^1118^ isogenic line (control). A four-way ANOVA revealed a significant genotype-by-treatment-by-age interaction effect on oxygen consumption rate (F_1,302_ = 5.65, *p* = 0.0180). In both panels, * *p* < 0.05, ** *p* < 0.01, *** *p* < 0.001, and **** *p* < 0.0001 after post hoc Tukey’s tests for multiple comparisons. Error bars represent standard errors.

To determine whether changes in Ance levels in specific tissues/organs are involved in the lisinopril effect on the whole-body metabolic rate, we next knocked down *Ance* in the muscle, Malpighian tubules, and nervous system using an RNA interference (RNAi) approach. We did not observe a statistically significant difference in metabolic rate between muscle-specific *Ance* knockdown flies (31.67 nmol/dL/mg body weight ±0.74) and control flies (31.40 nmol/dL/mg body weight ±0.80) or any significant change in metabolic rate between genotypes in response to lisinopril. On the other hand, our analysis showed significant genotype-by-treatment-by-sex and genotype-by-treatment-by-age interaction effects when *Ance* was knocked down in the Malpighian tubules and the nervous system, respectively (Figure 7).

Malpighian tubule-specific knockdown of *Ance* (*c42*-*Gal4* > *Ance*-RNAi) resulted in a significant decrease in oxygen consumption rate in both female and male flies, independent of age (Figure 7a). However, the effect of the drug showed sex-specific differences. Lisinopril treatment decreased the oxygen consumption rate further in *c42*-*Gal4* > *Ance*-RNAi females but had no effect in control female flies (Figure 7a). In contrast, the drug significantly reduced the oxygen consumption rate in control males, but no differences were observed between *c42*-*Gal4* > *Ance*-RNAi lisinopril-treated and untreated males (Figure 7a). The knockdown of *Ance* (*elav*-*Gal80* > *Ance*-RNAi) in the nervous system led to a significant increase in oxygen consumption rate compared to control flies only in young flies, independent of sex (Figure 7b). In addition, lisinopril significantly increased the oxygen consumption rate in young control flies, but not in *elav*-*Gal80* > *Ance*-RNAi flies (Figure 7b). Altogether, our findings suggest that lisinopril alters the whole-body metabolic rate in *D. melanogaster* through a reduction in *Ance* in the Malpighian tubules and in the nervous system, but in a sex- and age-specific manner.

**Figure 8:**
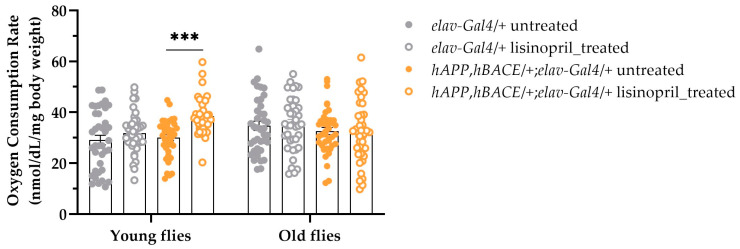
Lisinopril increases the whole-body metabolic rate of young flies with AD-like symptoms regardless of sex. A four-way ANOVA revealed a statistically significant effect of the genotype-by-treatment-by-age interaction term (F_1,301_ = 3.92, *p* = 0.0486) on metabolic rate. *** *p* < 0.001 after post hoc Tukey’s tests for multiple comparisons. Error bars represent standard errors.

Data suggest that ACEIs might delay the symptoms of neurodegeneration in AD in animal models [21,22]. Additionally, it has been reported that the reduced cerebral metabolic rate of glucose is normally found in the AD brain [23]. Based on these observations, we wanted to explore whether lisinopril might alter the whole-body metabolic rate of flies that we previously reported exhibiting AD-like symptoms [21]. As shown in Figure 8, we observed a statistically significant difference in metabolic rate between lisinopril-treated *hAPP*, *hBACE*/*+*; *elav-gal4*/*+* AD young flies and their matched untreated flies, but not in old flies. No differences were found between lisinopril-treated control *elav-gal4*/*+* flies and those untreated at either age.

Thus, lisinopril significantly enhances the whole-body metabolic rate in flies with AD-like symptoms, but only at a younger age.

## 3. Discussion

Experimental studies in rodent models suggest that ACEIs may have beneficial effects on resting metabolic rate (RMR), offering potential advantages beyond their primary use in managing hypertension [5,6]. The impact of ACEIs on RMR has also been observed in human clinical studies, where hypertensive overweight or obese women treated with ACEI or angiotensin blockers showed significantly lower RMR than untreated patients after accounting for body composition and physical activity [20]. Similar to these findings, in the present study, we report that the ACEI lisinopril influences the body-weight-adjusted metabolic rate in *D. melanogaster*, with the effect being independent of locomotor behavior. The cross-species consistency of the findings reinforces the notion that ACEIs play a role in modulating metabolic rate through evolutionarily conserved mechanisms, making research performed in *D. melanogaster* likely to be applied successfully to humans.

In our study, we further demonstrate significant inter-individual genetic variation in metabolic rate changes in response to lisinopril, with some DGRP lines having higher metabolic rates and others having lower metabolic rates. However, the genetic component is dependent on the sex and age of the flies. The findings agree with our previous work, showing that lisinopril perturbs the thoracic mitochondrial respiration rate and metabolic network structure of male flies in a genotype- and age-specific manner [16]. Additionally, they support emerging data in clinical studies revealing that the intersection between sex and age plays a crucial role in determining the effectiveness and safety of ACEIs on health outcomes [24,25]. Altogether, these observations highlight the need to collect better data on sex and age in studies that explore how genetic variations influence individual responses to ACEI therapy, if personalized treatment strategies are to be applied.

It has been reported that *Drosophila* Ance can convert Ang I to Ang II and inactivate bradykinin, even if these peptides are not present in *D. melanogaster* [14]. In this regard, one remarkable finding of this study is that when flies of the DGRP lines that showed the utmost change in metabolic rate in response to lisinopril were treated with the ARB losartan, we found that the direction of the effect of losartan on metabolic rate was the same as of the lisinopril effect in most of the lines. This result implies that the impact of lisinopril on metabolic rate in *D. melanogaster* can be, in part, attributed to the reduction in ancestral Ang II-equivalent signaling. To further support this idea is the observation in rodents that diet-induced obese *Agtr1a* knockout rats have a different metabolic rate than their wild-type counterparts [7].

Despite the observations made above, we also detected that whereas lisinopril induced a significant change in the metabolic rate of young DGRP-367 flies and old DGRP-808 flies, losartan did not do so, suggesting that lisinopril might influence the trait in these flies through the inhibition of bradykinin-like peptide degradation. Notably, young DGRP-367 and old DGRP-808 male flies treated with lisinopril not only had a higher metabolic rate than their untreated equivalents but were also more resistant to cold. In mammals, cold exposure activates the sympathetic nervous system (SNS), which is crucial for the body’s adaptation to cold [24]. The SNS triggers the release of catecholamines that stimulate thermogenesis, particularly in brown adipose tissue (BAT) [25]. This process generates heat to maintain core body temperature. Additionally, the SNS causes vasoconstriction in peripheral blood vessels, reducing heat loss through the skin [26]. This sympathetic response is essential for enhancing cold tolerance and ensuring survival in cold environments. Recently, one key study in male mice demonstrated that the reduced degradation of bradykinin induced by ACEIs resulted in enhanced BAT UCP1 expression and thermogenesis, as well as an increase in body temperature via B_2_ receptors [20]. Similar to the study in male mice, we found that, although there were no changes in the thoraces and abdomens, the expression of *Ucp4b* and *Ucp4c* was significantly increased in the heads of young DGRP-367 male flies following the lisinopril treatment. Several ectotherms increase their metabolic rates when acclimating to low temperatures [27]. This process, referred to as metabolic compensation, helps these organisms to maintain basic physiological functions under cold conditions [27]. It has been reported that the catecholamine dopamine plays a role in regulating metabolic rate and temperature sensitivity in *D. melanogaster* [28], and UCP4b and UCP4c are involved in cold thermogenesis [29,30,31,32]. As such, a potential explanation for the fact that lisinopril-treated young DGRP-367 male flies were more resistant to cold exposure and had increased metabolic rate is that dopamine and UCP-dependent thermogenesis are increased in the brains of the flies. Further research is, however, needed to corroborate this idea. To this end, experiments are currently underway in our laboratory to measure dopamine levels in the brain of lisinopril-treated young DGRP-367 male flies and their untreated counterparts, and to measure ATP production and proton leak in mitochondria isolated from the brains of these flies.

The involvement of the brain in the effect of lisinopril on whole-body metabolic rate is further supported by our RNAi experiments, showing that the increase in metabolic rate induced by lisinopril in young flies, regardless of sex, depends on Ance in the nervous system. In line with this finding, we also found that lisinopril increased metabolic rate in young flies with AD-like symptoms. A growing body of evidence argues for ACE overactivation playing a role in the development and progression of AD in humans [33,34]. This is in complete agreement with experimental work attributing neuroprotective properties to ACEIs in AD patients and AD animal models [21,22]. Interestingly, mechanisms underlying body temperature control are compromised in old age, a time when the incidence of AD rises [35]. A lower-than-average body temperature has been reported for those aged 60 years and older [36] due to impaired thermogenesis that may be partially attributed to reduced BAT activity, a characteristic of aging [37]. Given the lack of longitudinal studies, there is no consensus regarding changes in average body temperature with AD progression or whether the loss of thermoregulatory capacity precedes or follows AD symptoms [36]. However, evidence supports the idea of hypothalamic neurodegeneration triggering the loss of thermoregulation, creating a feedback loop that further contributes to brain dysfunction [36]. These observations, together with our findings, motivate additional studies to investigate whether lisinopril may have therapeutic potential to prevent or delay AD by ameliorating thermoregulation deficiencies in the elderly.

Depending on the genotype, sex, and age of the flies, we found that lisinopril treatment can also result in a significant reduction in metabolic rate. This is most likely due to the site of action of the drug and the impact that the drug has on fitness traits related to metabolic rate. In fact, a reduction in metabolic rate was also seen in flies, where *Ance* was knocked down in the Malpighian tubules, regardless of sex and age. Moreover, lisinopril significantly reduced the metabolic rate of male control flies but not of those with *Ance* knocked down in the Malpighian tubules, indicating that a lisinopril-induced reduction in metabolic rate depends on *Ance*. In mammals, Ang II produced by renal ACE is the key regulator of nephron sodium excretion [38]. Malpighian tubules are the main excretory organs in *D. melanogaster* [39]. Recent work revealed that the coordination between cellular energy demands and metabolic processes in *Drosophila* renal system delays premature aging by maintaining mitochondrial efficiency, reducing oxidative stress, promoting autophagy, and regulating key longevity pathways [40]. This coordination helps to protect renal cells from damage and dysfunction, ultimately contributing to better performance and longevity [40]. Thus, it is conceivable that lisinopril treatment may lead to a hypometabolic state as a result of alterations of the functional-metabolic coupling in the Malpighian tubules. The entry into a hypometabolic state can then provide flies with a greater chance to survive when facing extreme environmental or physiological stress [41].

In conclusion, while there is evidence from mammalian models that ACEIs might impact overall metabolic rate, comprehensive studies on this topic are relatively scarce. In the present study, we took advantage of *Drosophila*’s short life cycle and lifespan, as well as the array of genomic resources and genetic tools available to the scientific community, such as the DGRP and transgenic lines for tissue-specific expression, to offer new insights into this exciting area of research. We demonstrated that the effects of ACEIs on metabolic rate depend on the genetic background and sex of the individual and may be more pronounced in specific contexts, such as chronological age. We also established that the role of Ance in *Drosophila* as a modifier of metabolic rate varies between tissues and corroborated work in rodents, suggesting that local brain RAS plays a critical role in metabolic rate regulation [42]. Future work will involve using the full set of DGRP lines to identify specific genes mediating the changes in metabolic rate in response to lisinopril. This approach can help identify potential gene targets for more precise interventions in metabolic disorders.

## 4. Materials and Methods

### 4.1. D. melanogaster Strains and Husbandry

In this study, we used 22 wild-derived inbred lines from the DGRP that were obtained from the laboratory of Dr. Trudy Mackay at Clemson University. Virgin female and male flies of each of the 22 DGRP lines were either fed a cornmeal, agar, molasses, and yeast medium or received 1mM Lisinopril (AvaChem Scientific: #2589. San Antonio, TX, USA) from the day of eclosion until one week or five weeks of age. Virgin male and female flies from a subset of 11 DGRP lines were also fed 1mM Losartan (AvaChem Scientific: #1645. San Antonio, TX, USA).

For knockdown experiments, we obtained *UAS*-RNAi-*Ance* (*w^1118^*; P{GD5388}v41219, stock# v41219) and *w^1118^* (Stock# v60000) from the Vienna Stock Center (Vienna, Austria). The *c42*-*Gal4* (Stock# 30835) and *DJ667*-*Gal4* (Stock# 8171) were obtained from the Bloomington Stock Center (Bloomington, IN, USA). *Tub*-*Gal80^ts^*/*Cyo*; *elav*-*Gal4* was generated in the lab of Dr. Jumbo Lucioni using *Tub*-*Gal80^ts^*; *TM2*/*TM6B* (Stock# 7019) and *elav*-*Gal4* (Stock# 602358) from the Bloomington Stock Center. We crossed females from each driver and *UAS*-RNAi-*Ance* or *w^1118^* males to generate the organ system-specific *Ance* knockdown flies and the *w^1118^* control flies.

The *Drosophila* AD model flies (i.e., h*APP*, h*BACE*/+; *elav*-*Gal4*/+) and their control counterparts (i.e., *elav*-*Gal4*/+) are described in [21].

We reared stocks and experimental flies in culture vials, at a constant temperature of 25 °C, 60–75% relative humidity, and 12/12 h light/dark cycle. A temperature of 29 °C was used to rear AD flies and adult flies for RNAi experiments using the *elav*-*Gal80^ts^* transgene to permit GAL4 activity.

### 4.2. Metabolic Rate Assay

We first weighed each fly to 0.1 mg accuracy by keeping them on ice. Subsequently, we assessed the metabolic rate by monitoring the whole-body consumption rate of each fly using the Loligo Microplate (24-well plate) Respirometry System (Loligo^®^ Systems, Viborg, Denmark) at the University of Alabama at Birmingham Small Animal Phenotyping Core. The protocol details are reported in [43]. Briefly, oxygen concentration was measured in each well for 60 min, with the first 30 min of measurements excluded from the analysis to allow each fly to acclimate in a new environment and thereby minimize stress. While 10 individual flies per treatment/sex/age were tested for each DGRP line, 20 individual flies per genotype/treatment/sex/age were examined for the *Ance* knockdown and AD model experiments.

### 4.3. Climbing Assay

We evaluated climbing performance using the protocol previously described in [21]. Briefly, cohorts of 15–16 flies were transferred from standard medium into testing vials. Flies were then tapped to the bottom of the vial and given 10 s to pass an 8 cm mark. Five sets of experiments were performed, and data were expressed as the proportion of flies passing the 8 cm mark per genotype, sex, and treatment groups.

### 4.4. Chill-Coma Recovery Assay

We used time to recover from chill-coma to test cold tolerance. Groups of 25 flies for each genotype and sex were subjected to 0 °C for 3 h. Flies were then returned to room temperature and the time to restore locomotor function was measured. Five groups per genotype/treatment/sex/age were examined.

### 4.5. qPCR

We isolated total RNA using Invitrogen™ TRIzol™ Reagent (Thermo Fisher Scientific, Waltham, MA, USA). Isolated RNA was then used to make cDNA, using the High-Capacity cDNA Reverse Transcription kit (Thermo Fisher Scientific, Waltham, MA, USA). We performed qPCR using a Syber Green Master mix (Bio-Rad, Hercules, CA, USA) and 50 ng total of cDNA per reaction. We determined relative expression levels using the 2^−ΔΔCt^ formula [44] by normalizing *Ucp4b* and *Ucp4c* to the housekeeping genes *rp49* and *α-tubulin*. The sequence of primers used for qPCR was obtained from [32].

### 4.6. Statistics

We ran four- and three-way ANOVA models, as appropriate, to analyze our data and assess the main effects of genotype, drug treatment, sex, and age, and all possible interaction terms. In all models, we used the Tukey test for post hoc pairwise comparisons to determine significant differences between groups. A square root transformation was applied to CCRT data for old flies and metabolic rate data of the nervous system-specific *Ance* knockdown experiment to meet the ANOVA assumption of normality. Statistical analyses were performed with SAS version 9.4 (SAS Institute, Cary, NC, USA), and a significance level of 0.05 was used throughout the study.

## Figures and Tables

**Table 1:** Four-way ANOVA for metabolic rate in 22 DGRP lines.

Source ^a^	Df ^b^	MS ^c^	*F* ^d^	*p*-Value
Genotype	21	1130.73	0.80	0.6927
Treatment	1	14.97	0.01	0.9126
Sex	1	3817.85	38.76	**<0.0001**
Age	1	5764.66	6.27	**0.0206**
Genotype × Treatment	21	1212.95	2.88	**0.0106**
Genotype × Sex	21	98.51	0.22	0.9994
Genotype × Age	21	919.33	1.46	0.1757
Treatment × Sex	1	404.71	2.27	0.1471
Treatment × Age	1	26.89	0.07	0.7901
Sex × Age	1	257.96	0.66	0.4243
Genotype × Treatment × Sex	21	178.65	1.40	0.2240
Genotype × Treatment × Age	21	370.05	2.90	**0.0092**
Genotype × Sex × Age	21	388.68	3.04	**0.0069**
Sex × Treatment × Age	1	15.91	0.12	0.7275
Genotype × Treatment × Sex × Age	21	127.68	2.22	**0.0012**

^a^ Source of variation. ^b^ Degree of freedom. ^c^ Mean Squares computed from Type III Sums of Squares. ^d^ *F*-statistics based on the ratio of Mean Squares. *p*-values significant at less than 0.05 are highlighted in bold.

## Data Availability

The data that support the findings of this study are available upon request to the corresponding author.
